# The varied functions of the giant muscle scaffold protein obscurin

**DOI:** 10.3389/fcell.2025.1689326

**Published:** 2026-01-05

**Authors:** James Novac Di Paola, Frieder Schöck

**Affiliations:** Department of Biology, McGill University, Montreal, QC, Canada

**Keywords:** obscurin, sarcomere structure, myofibrillogenesis, M-line, ankyrin, myosin, titin, RhoGEF domain

## Abstract

Obscurin is a giant protein encoded by the *OBSCN* gene in human myocytes, known for its roles in sarcomere organization, elasticity, stretch response, and myofibrillogenesis. Alternative splicing of *OBSCN* generates variants with distinct properties and localizations, including small kinase variants in mammals. Investigating obscurin-like 1 (Obsl1) and striated muscle-enriched protein (SPEG), the other members of the obscurin family in higher eukaryotes, has allowed a better understanding of obscurin family function in the sarcoplasmic reticulum, mitochondrial fragmentation, and kinase domain regulation. Obscurin’s association with ankyrin isoforms in vertebrates demonstrates participation in membrane and cytoskeletal organization within muscle tissues while binding to myosin actively contributes to the formation and maintenance of the sarcomeric contractile apparatus and the M-line. The Rho-guanine nucleotide exchange factor (RhoGEF) domain of obscurin suggests a role in activating small GTPases and autoregulation. Obscurin also binds titin, indicating a dynamic function in monitoring sarcomere extension and relaying cues in muscle remodeling. Obscurin and titin can further form a tertiary complex with myomesin in vertebrates, reinforcing its importance in M-line assembly and sarcomeric organization. Beyond muscle tissue, obscurin is expressed and plays additional roles in various other organs, including skin, brain, kidney, liver, spleen, and lung. Potential tumor-suppressing properties have been revealed through *OBSCN* lncRNAs and epigenetic regulation. This review aims to provide a comprehensive overview of obscurin’s molecular functions and interactions by discussing the effects of its differential expression and its interactions with binding partners, along with the differences and similarities between vertebrate and invertebrate obscurin.

## Introduction

Obscurin is a large modular protein involved in structural and signaling functions. Because obscurin is found in all animal musculature, we discuss differences and especially similarities in obscurin function between vertebrates and invertebrates to reveal evolutionarily conserved functions shared by all animals.

In vertebrates, obscurin was initially characterized as a novel binding partner of titin in a yeast two-hybrid screen (Young et al., 2001). It was named after the adjective “obscure” due to the difficulty of its characterization, owing to its complex structure and considerable size (Young et al., 2001). Further research has revealed obscurin’s binding partners and functional properties within striated muscle tissue (Young et al., 2001; Kontrogianni-Konstantopoulos et al., 2009; Fukuzawa et al., 2008). Similar to titin, the prototypical isoforms of obscurin are giant proteins abundant in myocytes, primarily known for contributing to sarcomere organization, elasticity, stretch response, and myofibrillogenesis (Kontrogianni-Konstantopoulos et al., 2009). Despite some structural similarities to titin, obscurin has a distinct subcellular localization pattern, acting primarily at the M-line (Fukuzawa et al., 2008), whereas titin spans up to half of the sarcomere and performs different functions at the Z-disc and I-band, as well as the A-band and, in some organisms, the M-line (Noureddine and Gehmlich, 2023).

The first obscurin, called UNC-89, was identified in a genetic screen for disorganized sarcomeres in *C. elegans* (Waterston et al., 1980) and subsequently characterized at the molecular level (Small et al., 2004; Benian et al., 1996). In *C. elegans*, *Drosophila*, and other invertebrates, a single gene is alternatively spliced, generating multiple variants with distinct properties and localizations (Small et al., 2004; Ferrara et al., 2005). In higher eukaryotes, gene duplication and rearrangement events have led to an expansion of the obscurin family to three members: obscurin, obscurin-like 1 (Obsl1), and striated muscle-enriched protein (SPEG) (Blondelle et al., 2019; Agrawal et al., 2014). Their discovery has enabled a better understanding of the obscurin family function within the sarcoplasmic reticulum (SR), mitochondria, and the regulation of the kinase domain (Blondelle et al., 2019; Agrawal et al., 2014). Vertebrate obscurin association with ankyrin isoforms in muscle tissue shows its role in membrane and cytoskeletal organization (Raeker and Russell, 2011), while its Rho-guanine nucleotide exchange factor (RhoGEF) domain links it to the activation of small GTPases and autoregulation (Ford-Speelman et al., 2009; Koch et al., 2023). Obscurin in non-muscle tissues plays additional roles beyond sarcomere organization (Kontrogianni-Konstantopoulos et al., 2009; Ackermann et al., 2014; Kontrogianni-Konstantopoulos et al., 2003). Potential tumor-suppressing properties were also revealed by *OBSCN* long non-coding RNAs (lncRNAs) and epigenetic regulation (Guardia et al., 2021).

This review aims to provide a comprehensive overview of obscurin’s structure and function by discussing the impacts of its differential expression and association with binding partners while briefly covering its functions within non-muscle tissues. One main focus will be on the differences and similarities between invertebrate and vertebrate obscurin.

## Overview of muscle tissue constituents

**FIGURE 1:**
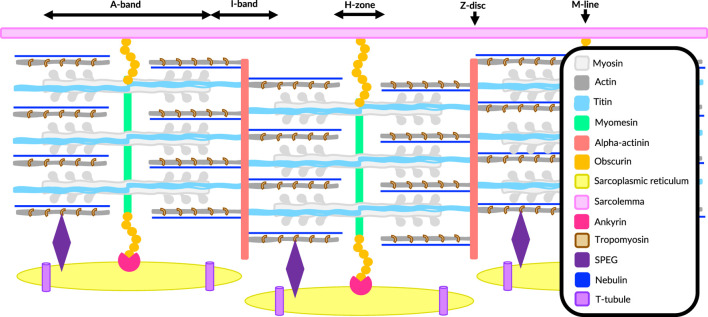
Cartoon of the vertebrate sarcomere in skeletal muscle.

The sarcomeric cytoskeleton is a structural component within skeletal muscle cells, organized into bundles of myofibrils (Lange et al., 2020). The sarcomeric center, called the A-band, is occupied by thick myosin filaments, while the surrounding region, which contains only thin actin filaments, is referred to as the I-band (Lange et al., 2020). The M-line, where obscurin preferentially localizes, crosslinks myosin filaments at the sarcomeric center, while the Z-disc anchors the overlapping antiparallel actin filaments at either end of the sarcomere (Figure 1) (Lange et al., 2020). The overlapping actin and myosin filaments within the sarcomere enable contraction (Lange et al., 2020).

The distance between the M-line and Z-disc is spanned by titin in vertebrates, which anchors its N-terminal in the Z-disc and its C-terminal in the M-line via links with myomesin and obscurin (Gautel and Djinovic-Carugo, 2016). However, the insect titin homolog Sallimus (Sls) in *Drosophila* and the nematode titin homolog TTN-1 in *C. elegans*, although ∼2 MDa in size, are too short to span the I-band and reach obscurin at the M-line, and thus the titin-like molecules projectin in insects and twitchin in nematodes help bridge the gap in the A-band without connecting to obscurin (Schueder et al., 2023; Forbes et al., 2010).

Titin and the giant modular protein nebulin also act as scaffolding agents, providing attachment sites for other sarcomeric elements to regulate their physical orientation (Kontrogianni-Konstantopoulos et al., 2009; Gautel and Djinovic-Carugo, 2016) They are both integral components of the sarcomere itself in all animals, whereas obscurin is an integral myofibril component in insects and nematodes (Small et al., 2004; Benian et al., 1996) but is restricted to the myofibril surface in vertebrates (Kontrogianni-Konstantopoulos et al., 2003; Manring et al., 2017; Katzemich et al., 2015). Vertebrates also have ankyrin proteins that can link sarcomere structures to the SR via obscurin, while a similar connection to calcium reservoirs is mediated by SPEG (Luo et al., 2021; Perry et al., 2013). Structurally, titin and obscurin are multidomain proteins mainly composed of immunoglobulin (Ig) and fibronectin type III (FnIII)-like domains (Meyer and Wright, 2013).

## Obscurin evolution

**FIGURE 2:**
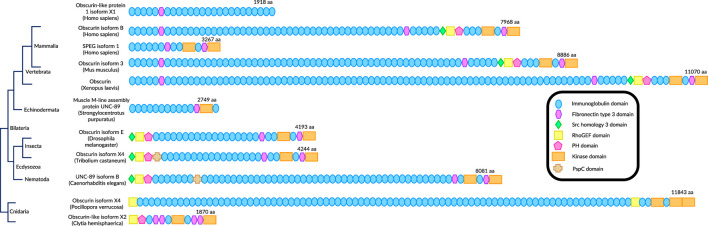
Evolutionary conservation of obscurin homologs in animals. Proteins are not to scale as only conserved domains are shown.

Obscurin binds to cytoskeletal structures in muscle and non-muscle cells in all organisms analyzed, suggesting that the currently unidentified myosin-binding domain(s) of obscurin predates muscle evolution (Manring et al., 2017; Steinmetz et al., 2012). Nevertheless, the canonical obscurin domain organization of a RhoGEF domain, followed by several Ig and FnIII and two C-terminal kinase domains, is first observed in Cnidaria, suggesting that obscurin arose together with striated muscle evolution (Figure 2). From there, genomic rearrangements likely gave rise to the two predominant types of obscurins in Bilateria: the ecdysozoan obscurin, found in nematodes and insects with the RhoGEF domain at the N-terminus and many intervening Ig domains, versus the chordate obscurins, with Ig domains inserted before the RhoGEF domain. Finally, gene duplication created three orthologs in vertebrates: obscurin, Obsl1, and SPEG (Figure 2).

## Obscurin isoforms

### Alternative splicing

Katzemich et al. (2012) identified four obscurin isoforms in the thorax of mature *Drosophila*, one of which is the largest prevalent isoform expressed in the indirect flight muscles and another being a smaller isoform uniquely found in larvae. All isoforms contain the Ig14–16 and kinase 1 domains, suggesting that alternative splicing predominantly affects the N-terminus (Katzemich et al., 2012). Mass spectroscopy analysis of pupae suggests that variants are generated by gene splicing instead of proteolytic cleavage (Katzemich et al., 2012). Similar to *Drosophila*, *C. elegans* UNC-89 is a complex gene that uses three promoters and extensive alternative splicing to generate at least 16 polypeptides ranging in size from 156 to 900 kDa, some of which are expressed in distinct muscle types (Small et al., 2004; Benian et al., 1996; Ferrara et al., 2005). UNC-89 and *Drosophila* obscurin lack ankyrin-binding and IQ domains that are found in mammalian species (Manring et al., 2017; Katzemich et al., 2015; Katzemich et al., 2012).

Many isoforms can arise from the 119 exons of *OBSCN* due to the modular assembly of its Ig and FnIII domains, which are generally expressed through one domain per exon (Young et al., 2001; Perry et al., 2013). The Ig tandem repeat region of *OBSCN* encodes an additional 10 exons for Ig motifs that are not included in obscurin A (Young et al., 2001). Evidence of differential splicing among obscurin isoforms was also observed in immunoblot studies targeting specific obscurin epitopes, revealing immunoreactive bands of various lengths corresponding to obscurin splice variants (Perry et al., 2013). Ackermann et al. (2014) also conducted a systematic investigation of obscurin expression profiles, showing that various obscurin isoforms ranging in size from ∼50 to ∼970 kDa localize to both muscle and non-muscle tissues, where they exhibit nuclear, cytosolic, and membrane distributions. Two novel obscurin isoforms of 110 and 120 kDa in length were detected in the nucleus of epithelial cells, the former containing the RhoGEF domain and the latter containing kinase domains (Perry et al., 2012).

Skeletal obscurin variants in vertebrates exhibit differential localization: isoform A is mainly found at the M-line; isoform B at the A/I junction; and smaller isoforms at the Z-discs and Z/I junctions (Bowman et al., 2007).

A recent study found widespread regulation of exon inclusion at multiple sites around the 5′ end, central part, and 3′ end of the human *OBSCN* gene during cardiac and skeletal myogenesis (Oghabian et al., 2025). Oghabian et al. (2025) suggested that differential inclusion during skeletal muscle development stems from altered Bub3 regulation of alternative splicing as Bub3 expression is reported to be higher in fetal than in postnatal skeletal muscles. Bub3 is an essential mitotic regulator that interacts with the splicing machinery in the interphase nucleus, and its silencing enhances exon skipping in human foreskin fibroblasts (Wan et al., 2015). Variations in the last exon of *OBSCN* were noted to produce isoforms of different lengths when comparing postnatal muscle (Oghabian et al., 2025). Further investigations into the regulation of Bub3-mediated alternative splicing in maturing postnatal muscle may reveal additional stage-specific patterns with broader functional properties and binding partners.

### Obscurin isoform A

Obscurin A is the prototypical member of its family, containing 54 Ig repeats and 2 FnIII domains mainly arranged in tandem at the N-terminal, followed by several signaling domains: src homology 3 (SH3), IQ, RhoGEF, and pleckstrin homology (PH) domains (Young et al., 2001; Kontrogianni-Konstantopoulos et al., 2009). An IQ domain suggests that this variant binds calmodulin and calmodulin-like proteins, while RhoGEF and PH tandem domains suggest its potential function in RhoGTPase-regulated signaling cascades (Meyer and Wright, 2013). The C-terminal end of obscurin A contains two tandem Ig domains and a non-modular span of approximately 400 amino acids holding several consensus phosphorylation motifs for extracellular signal-regulated kinases (ERKs), such as those found in the N-terminal portion of titin (Young et al., 2001). As elastic properties are bestowed on titin by its semi-flexible Ig tandem repeats (Bang et al., 2001), obscurin’s architecture, also predominant in Ig repeats, might mitigate strain from repeated contraction and stretching of the myofibrils. This mitigation could induce tension in surrounding muscles while reducing destructive force through conformational changes (Meyer and Wright, 2013). As the C-terminal region of obscurin A interacts with small ankyrin-1 (sAnk1, also called sAnk1.5) at the M-line in skeletal muscle (Busby et al., 2011), this protein might convey additional information in a tension-sensitive manner through the modulation of SR intracellular calcium levels.

### Obscurin isoform B

Obscurin B (∼870 kDa in humans) is an alternate isoform similar to obscurin A in structure without the non-modular C-terminal region, instead possessing two serine-threonine kinase domains (SK1-2) that share homology with the titin family of myosin light chain kinases (Russell et al., 2002). Splicing at the 3′ end of the *OBSCN* gene also adds an adjacent FnIII domain and two Ig repeats (Fukuzawa et al., 2005).

A study of rat obscurin B showed that it preferentially localizes at contact sites in developing ventricular cardiac myocytes and is regulated by calcium levels (Wang et al., 2024). An insulin treatment of embryonic cardiomyocytes exhibited rapid N-cadherin phosphorylation at Ser-788, and phosphoproteomics analysis confirmed that the juxtamembrane section of the N-cadherin cytoplasmic domain is a target of the obscurin B SK1 domain (Wang et al., 2024). Obscurin B appears to integrate both metabolic and ionic signals (Wang et al., 2024); thus, further research could determine its role in cardiomyocyte growth and adhesion and in heart conditions such as dilated cardiomyopathy. Wang et al. (2024) postulated that obscurin SK1 phosphorylation of N-cadherin at Ser-788 limits N-cadherin and p120-catenin binding, thereby negatively regulating RhoA activity, which is implicated in cell adhesion.

### Smaller isoforms and variants

The SK1 and SK2 domains of obscurin can be present in two shorter, alternatively spliced kinase isoforms containing either only the SK1 domain, termed single kinase isoforms, or partial SK1 and SK2 domains, termed tandem kinase isoforms (Borisov et al., 2008). Hu and Kontrogianni-Konstantopoulos (2013) demonstrated that both kinase domains exhibit enzymatic functions and can undergo autophosphorylation. They determined that SK2 efficiently and specifically phosphorylates the cytoplasmic domain of N-cadherin, which is involved in cell adhesion (Hu and Kontrogianni-Konstantopoulos, 2013). They also found that SK1 binds to, but does not phosphorylate, the extracellular domain of the Na+/K + -ATPase pump, NKAβ1, which modulates intercellular adhesion (Hu and Kontrogianni-Konstantopoulos, 2013). It is suggested that obscurin’s kinase domains are enzymatically active and likely mediate cellular adhesion through interactions with various substrates and ligands, both within cardiac and skeletal muscle and potentially across other tissues (Hu and Kontrogianni-Konstantopoulos, 2013).

## Obscurin, Obsl1, and SPEG

### Impact of modified obscurin expression in animal models

In *C. elegans*, a loss-of-function mutation in UNC-89 results in reduced whole-animal motility, disorganized sarcomeres, and the absence of M-lines (Waterston et al., 1980; Small et al., 2004; Benian et al., 1999). UNC-89 mutants display disorganized thick filaments by immunostaining (Qadota et al., 2008a; Wilson et al., 2012), although in early larvae, sarcomeres are normally organized (Spooner et al., 2012). This suggests that UNC-89 is required for the maintenance or growth of sarcomeres rather than their initial assembly. In *Drosophila*, obscurin participates in the development of a symmetrical and functional sarcomere in the indirect flight muscle, mostly with regard to the formation of M-lines and the proper alignment of thick filaments (Katzemich et al., 2012). Obscurin depletion experiments in zebrafish indicate its role in myofibrillogenesis, particularly in mediating integrin-extracellular matrix interactions (Raeker and Russell, 2011).

More recent obscurin knockout studies in mice show a reduction in Ca^2+^ release from the SR in depolarized skeletal muscle fibers, indicative of obscurin’s potential involvement in preserving SR structure (Pierantozzi et al., 2022). Immunoreactivity for CD45 and type III collagen in obscurin knockout mice showed increased connective tissue, leukocyte infiltration, and hypercontractures in several muscle fibers following heavy exercise due to disordered M-lines, H-zones, and A-bands, most of which were observed in the diaphragm (Randazzo et al., 2017). Lower tolerance to mechanical stress was noted in aging obscurin knockout mice (Randazzo et al., 2017). Excessive muscle activity, accompanied by heavy breathing, explains the prominent alterations in the diaphragm, while other myocytes at different locations may perform compensatory functions provided by Obsl1 (Geisler et al., 2007). These findings indicate that obscurin has functions beyond ensuring sarcomere structure, such as modulating the activity of calcium-handling proteins and providing the spatial distribution for efficient electromechanical coupling.

Truncated variants of *OBSCN* were documented to induce episodes of rhabdomyolysis and exercise intolerance in myofibers from human subjects (Zemorshidi et al., 2024). Decreased Ca^2+^ in the SR of human patients with myoblasts containing bi-allelic loss-of-function variants in *OBSCN* was also noted to predispose to starvation (Cabrera-Serrano et al., 2022).

### Redundancy between obscurin and Obsl1

Obsl1 knockout mice displayed a comparable phenotype to double Obsl1 and obscurin knockout mice (dKO) regarding sarcolemma integrity and the assembly and stability of the dystrophin–sarcoglycan complex in skeletal muscle (Blondelle et al., 2019). Muscle cross-sections showed no difference in the number of centrally located nuclei, indicating that both knockout types support active regeneration and suggesting that other proteins contribute to functional compensation for myofibrillogenesis and sarcomere structure (Blondelle et al., 2019). Only obscurin knockout mice showed a significant decrease in sAnk1 levels due to increased protein recycling (Blondelle et al., 2019). While Obsl1 KO had little effect, SR calcium-handling alterations were predominantly observed in dKO skeletal muscle cells, with decreased sarcalumenin and sarcoplasmic reticulum calcium-ATPase (SERCA) expression and increased Casq2 and junctional SR proteins such as triadin, junctin, and RyRs (Blondelle et al., 2019).

Fujita et al. (2025) linked diastolic dysfunction and heart failure to dKO mice in cardiomyocytes, which were further characterized by decreased lifespan, mitochondrial dysfunction, altered metabolism, and autophagy. The loss of obscurin alone similarly caused impaired SR function and decreased intracellular calcium reuptake, whereas the loss of Obsl1 was associated with mitochondrial fragmentation (Fujita et al., 2025). Increased levels of dynamin-related protein 1 (Drp1), which regulates mitochondrial fission, were also associated with the loss of Chchd3, which reportedly binds Obsl1 for mitochondrial cristae formation (Fujita et al., 2025). Fujita et al. (2025) observed compromised cristae architecture and reformed mitochondrial fission in dKO mice with an increased ratio of phosphorylated to total Drp1 levels. The loss of only Obsl1 resulted in stable Chchd3 and Drp1 levels (Fujita et al., 2025), which reveals functional redundancy and the involvement of obscurin in mitochondrial fragmentation.

### Mitochondrial fragmentation

In mice, dKO skeletal myocytes were found to downregulate mitochondrial electron transport chain proteins, in addition to muscle glycogen phosphorylase and GAPDH, which are responsible for glycogen breakdown and glycolysis (Blondelle et al., 2019). Reduced mitochondrial complexes I, II, III, IV, and V, along with monoamine oxidase A and B levels, were observed in tibialis anterior muscles (Blondelle et al., 2019). Reactive oxygen species, peroxiredoxin, and Sod2 were also elevated in response to insufficient mitochondrial development, suggesting increased oxidative stress (Blondelle et al., 2019).

In *C. elegans*, CRISPR/Cas9 was used to create UNC-89 mutants by inactivating its ATP-binding pocket in PK2 (Matsunaga et al., 2024). While sarcomere structure, force generation, and muscle function were normal in these mutants, fragmented mitochondria were observed, with increased and decreased basal respiration for complexes I and II, respectively, neither of which could be uncoupled (Matsunaga et al., 2024). This indicates that mutant mitochondria are already uncoupled. The uncoupling might result from the observed increase in the single uncoupling protein in the worm, UCP-4. Increases in glycolysis and ATP production might compensate for altered mitochondrial function and observed uncoupling as mutations significantly increased 6-phosphofructose kinase levels (Matsunaga et al., 2024).

Matsunaga et al. (2024) suggested that the mitochondrial fragmentation results from the observed increase in mitochondrial association of Drp1, which is known in multiple organisms to be regulated by phosphorylation (Matsunaga et al., 2024). This means that Drp1 may be a direct or indirect substrate of the PK2 kinase. The study indicates that mitochondria might receive signaling from the sarcomere via the PK2 domain of UNC-89 to match energy consumption and production (Matsunaga et al., 2024). Such signaling is plausible as sarcomere contraction in striated muscle requires substantial ATP supplied by mitochondria. Fragmented mitochondria may result from a lack of myofibril signaling via obscurin.

Communication between myofibrils and mitochondria during muscle development has been demonstrated in *Drosophila* to alter mitochondrial structure according to muscle type (Avellaneda et al., 2021). Indirect flight muscle mitochondria are elongated along the myofibril axis, whereas leg muscle mitochondria form laterally oriented tubular networks unassociated with myofibrils and are concentrated both centrally and peripherally within the muscle fiber (Avellaneda et al., 2021). Mutations of Drp1 were found to alter mitochondrial differentiation, converting the mitochondrial phenotype of leg muscles to that of the indirect flight muscle mitochondria (Avellaneda et al., 2021). However, the role of *Drosophila* obscurin in mitochondrial function has not yet been demonstrated.

### Kinase/interkinase domains of obscurin and SPEG

The kinase domains of *C. elegans* UNC-89 are both capable of binding small CTD phosphatase-like-1 (SCPL-1) (Qadota et al., 2008b). SCPL-1 knockdown by RNAi showed a mild defect in egg-laying muscles, and SCPL-1 was shown to be epistatic to UNC-89, suggesting that UNC-89 functions upstream of SCPL-1 in egg-laying muscles (Qadota et al., 2008b). Xiong et al. (2009) found that an UNC-89 kinase domain forms a ternary complex at the M-line with LIM-9 and SCPL-1. The interaction with LIM-9 indicates that UNC-89 is linked, through a network of bridging proteins, to the integrin-associated M-line costamere and, thus, to the extracellular matrix (Xiong et al., 2009). UNC-89 domains Ig1–Ig3 were also found to interact with copine domain protein atypical-1 (CPNA-1) located at both M-lines and dense bodies (Z-discs) (Warner et al., 2013). CPNA-1 was necessary for proper integration of UNC-89 into integrin adhesion complexes, thereby providing another association between UNC-89 and these structures (Warner et al., 2013). These studies indicate the potential role of obscurin kinase domains in various muscle-associated functions, including linking the sarcomere contractile apparatus with the membrane network.

Moreover, the portion of UNC-89, Ig53-Fn2, which resides just N-terminal to PK2, interacts with PPTR-2, a regulatory subunit of protein phosphatase 2S (Qadota et al., 2018). PPTR-2 co-localizes with UNC-89 at the M-line, and loss-of-function of PPTR-2 results in sarcomere disorganization when its paralog, PPTR-1, is also deficient (Qadota et al., 2018). The substrates for SCPL-1 and PP2A at the M-line are unknown, but given their association with the UNC-89 kinase domains, this suggests that these phosphatases and kinases may share substrates.

The first kinase domain of obscurin was further studied in *Drosophila* obscurin, leading to its atypical identification as a pseudokinase with mechanosensory properties that no longer binds calmodulin (Zacharchenko et al., 2023). It can bind ATP in the absence of magnesium, yet no ATPase, autophosphorylation, or phosphotransferase activity was detected (Zacharchenko et al., 2023). Zacharchenko et al. (2023) found that inactive pseudokinases are known to act as scaffolds, all while considering that this kinase might allosterically regulate the second one via mechanosensing properties residing within the interkinase domain.

The sequence between both kinase domains of UNC-89 is referred to as the interkinase domain, containing a disordered region of 647–742 amino acids, along with an Ig domain and an Fn3 domain (Qadota et al., 2020). Subjecting this interkinase region to single-molecule force spectroscopy revealed that it is elastic and acts as an entropic spring (Qadota et al., 2020). A portion of this region was subjected to an in-frame deletion by CRISPR/Cas9, which resulted in disorganization of the sarcomere structure, malfunctioning locomotion, and difficulty generating muscle force as sarcomeres no longer had continuous parallel M-lines or A-bands (Qadota et al., 2020). Additionally, when the CRISPR-generated in-frame deletion of UNC-89 was tagged with HA at its N-terminus, its expression was reduced by half, resulting in smaller body diameters, yet body wall muscles showed normally organized sarcomeres (Qadota et al., 2020). Accordingly, the interkinase domain must contribute toward sarcomere architecture through providing elasticity, notably in defining the spacing between adjacent M-lines and A-bands.

Sites within the interkinase domain were identified by comparing obscurin with its homolog SPEG, which also contains two similar kinase domains, SPEG-K1 and -K2 (Sutter et al., 2004; Fleming et al., 2021). Cardiac SPEG-K2 activity regulates SERCA2 phosphorylation for Ca^2+^ reuptake into the ER, and its deletion in mice caused cardiomyopathy (Quan et al., 2019). Fleming et al. (2021) found no major phosphorylation sites within catalytic domains of either obscurin kinase 1 or SPEG-K1. However, phosphorylation sites in the corresponding interkinase domains of obscurin kinase 1 indicate its dependence on autophosphorylation as sites detected in the wild-type were absent in a kinase-dead mutant (Fleming et al., 2021). The interkinase domain of obscurin might regulate PK1 activity through autophosphorylation, in addition to providing elasticity to maintain its optimal structure.

## Ankyrin isoforms and obscurin

### Obscurin and small Ankyrin-1 interaction

A known binding partner of obscurin is the splice isoform sAnk1 of the *ANK1* gene, an essential part of the SR network (Perry et al., 2013). Two distinct binding sites for sAnk1 in skeletal muscle were identified at the non-modular C-terminus of obscurin A: the first between amino acid residues 6,236–6,260 and the second between residues 6,312–6,360 (Armani et al., 2006). A novel obscurin-binding transcript called sAnk1.9 was also discovered, encoding the same amino acid sequences for the binding to the obscurin C-terminus (Armani et al., 2006). Both sAnk1 and sAnk1.9 were found to bind sites on obscurin’s C-terminus with comparable efficiency (Armani et al., 2006).

The direct interaction of obscurin and sAnk1 was the first link discovered between the contractile mechanism of the sarcomere and the SR membrane system. The network compartment of the SR aligns with M-lines and Z-discs on each sarcomere’s surface and is vital for Ca^2+^ homeostasis during the initiation of muscle contraction (Perry et al., 2013). In striated muscles, sAnk1 is the most abundant small ankyrin-1 isoform, mainly concentrated within the SR near M-lines, although it can also be located at Z-discs (Quan et al., 2019). An experiment with an sAnk1 mutant lacking the obscurin-binding site showed that it remained dispersed throughout the SR rather than localizing near the M-line (Li et al., 2025). siRNA-induced knockdown of obscurin similarly resulted in disorganized sAnk1 distribution within the SR and a failure of the SR to organize around the contractile apparatus (Borzok et al., 2007). Obscurin A depletion in zebrafish embryos resulted in disrupted SR patterning and inconsistent physical association with myofibrils (Agrawal et al., 2014). Furthermore, mice remain less affected during movement when lacking sAnk1 compared to obscurin knockout models, possibly because sAnk1 stabilizes the SR through obscurin association and because obscurin interacts with other ankyrin isoforms (Blondelle et al., 2019; Pierantozzi et al., 2022). Collectively, these studies support the model where the non-modular C-terminus of obscurin, which localizes at M-lines, functions to directly anchor sAnk1 and related isoforms, thereby helping tether the SR to the sarcomeric cytoskeleton. The role of obscurin in linking the sarcomere to the SR is conserved in *C. elegans*. Genetic analysis indicates that UNC-89 is essential for normal SR organization, calcium signaling, and muscle activity (Wilson et al., 2012). However, it remains unknown whether small ankyrins are involved in this linkage in nematodes.

Using the F3 fragment of obscurin, which encompasses only interacting sites for sAnk1, a recent *in vitro* study in skeletal muscle demonstrated that the hydrophobic residues of sAnk1 near the positively charged R64 to K73 region critical for obscurin binding are also important for interacting with SERCA1 (Li et al., 2025; Borzok et al., 2007). Li et al. (2025) proposed that sAnk1 interacts with both these proteins simultaneously, using opposing sides of the same β-sheet, to regulate SERCA1 activity during contraction and relaxation.

### Obscurin and ankyrin B interaction

Ankyrin B (AnkB) is another muscle-specific ankyrin isoform that relies on obscurin to function in sarcolemmal organization (Kontrogianni-Konstantopoulos et al., 2009). Encoded by *Ank2*, AnkB possesses obscurin-binding sites homologous to those of sAnk1 and sAnk1.9 (Kontrogianni-Konstantopoulos et al., 2009). Unlike sAnk1, which is concentrated in the network SR, AnkB localizes at the subsarcolemmal level near M-lines, similarly to obscurin (Randazzo et al., 2013). Two AnkB splice variants containing obscurin-binding domains were found to interact directly with obscurin in cardiac myocytes and thereby target the key cardiac signaling protein, PPA2, to the M-line (Cunha and Mohler, 2008).

In skeletal muscle tissue, Randazzo et al. (2013) demonstrated that obscurin knockout mice had disorganized localization of AnkB, showing the importance of obscurin-binding in targeting AnkB to the M-line. As AnkB is necessary for the subsarcolemmal concentration of dystrophin, obscurin depletion was also shown to impair dystrophin localization and thus cause aberrant organization of subsarcolemmal microtubules (Randazzo et al., 2013). These obscurin knockout mice showed impairment of sarcolemmal integrity and reduced muscle strength and function (Randazzo et al., 2013). Considering that obscurin-depleted zebrafish embryos also possess myofibrils with reduced association with the sarcolemma (Raeker and Russell, 2011), it is evident that obscurin’s interactions with sarcolemmal components such as AnkB are essential for the organization and tethering of the sarcolemma.

### Obscurin and AnkG107 interaction

Obscurin interactions were also observed with the muscle-specific ankyrin-G isoform, AnkG107, in cardiac muscle (Subramaniam et al., 2022). Knocking out obscurin allowed recognition of its role in complex formation between sAnk1 and AnkG107 through two ankyrin binding domains in the C-terminal domain of obscurin A (Subramaniam et al., 2022). Though the role of AnkG107 in cellular processes is still not fully established, Subramaniam et al. (2022) suggested that it allows complex formation as a means of sAnk1 ubiquitination and proteostasis regulation at the M-line, suggesting that obscurin acts as a dynamic scaffold during muscle repair and recycling.

## Obscurin and myosin

The interaction between obscurin and a vertebrate-specific novel isoform of myosin-binding protein-C, sMyBP-C slow variant-1, has been implicated in the assembly of M-lines and A-bands in skeletal muscle tissue (Ackermann et al., 2009). This variant features a unique C-terminus comprised of 26 new amino acid residues following the last Ig domain (C10), which remains conserved across all members of the MyBP-C slow family (Ackermann et al., 2009). Ackermann et al. (2009) found that the extreme N-terminal Ig2 domain of obscurin and the C10 domain of MyBP-C slow contain the minimal binding sites required for their direct interaction. The binding is notably strengthened by the presence of the 26 novel residues at the C-terminus of variant-1 (Ackermann et al., 2009). This group demonstrated that adenoviral-mediated overexpression of the Ig2 domain of obscurin, or its siRNA-mediated depletion, significantly disrupts the assembly of M-lines and the organization of myosin filaments into A-bands and impairs the proper localization of MyBP-C slow variant-1 (Ackermann et al., 2009). These findings suggest that the assembly of M-lines and myosin filaments into A-bands may, in part, be mediated through the specific interaction between obscurin and MyBP-C slow variant-1. This highlights the vital role of the N-terminus of obscurin and its interaction with ligands in the formation and maintenance of the sarcomeric contractile apparatus.

To examine interactions between myosin A and M-line proteins in muscle cells of *C. elegans*, chimeric myosin A mutants with 169 residues of the C-terminal rod showed efficient co-localization of UNC-89 and the zinc-finger protein UNC-98 at the M-line (Almuhanna et al., 2024). In contrast, myosin A mutants lacking these residues produced displaced filaments and showed only UNC-89 recruitment (Almuhanna et al., 2024). The same study also generated UNC-98/ZnF null mutants and found that UNC-89 was delocalized within a disorganized contractile apparatus (Almuhanna et al., 2024). These findings suggest that more than one site of the rod domain of myosin A is associated with the correct positioning of M-line proteins and that UNC-98/ZnF allows recruitment of UNC-89 to myosin A (Almuhanna et al., 2024). Myosin A was further characterized as organizing UNC-89 during embryonic assembly of the contractile apparatus as myosin A mutant embryos showed disrupted UNC-89, arrested elongation, and an inability to contract (Almuhanna et al., 2024). As in vertebrates, nematode UNC-89 is an integral member linking the contractile machinery to the M-line.

## Obscurin, titin, and myomesin complexes

### Obscurin and titin interaction

The physical interaction between obscurin and the sarcomeric giant protein titin has been well characterized as obscurin was identified in vertebrates through a screen for novel binding partners of the peripheral Z-disc region of titin (Young et al., 2001), likely reflecting an interaction during myofibrillogenesis as mature obscurin localizes predominantly near the M-line. That study found the Ig58/59 domains of obscurin interacting with the Z9/10 Ig domains of titin, suggesting that these titin domains function as a Z-disc targeting signal for the localization of certain obscurin isoforms. Separating either the titin or obscurin Ig domains abolished the interaction between these sites, indicating that tandem organization of these domains is necessary for functional binding (Young et al., 2001).

Subsequent work identified a distinct interaction between obscurin and a truncated titin isoform, novex-3, which is ∼700 kDa long and localizes at the I-band (Bang et al., 2001). The Ig58/59 region of obscurin was found to localize exclusively near the Z-disc when expressed as a GFP fusion protein in rat cardiomyocytes, with its distance to the Z-disc increasing proportionally in response to sarcomeric stretching (Bang et al., 2001), indicating that this obscurin isoform either expands or re-localizes dynamically in response to sarcomeric lengthening. It also shows that the novex-3 titin/obscurin complex may have strain-induced signaling properties, which the authors propose may help trigger sarcomeric restructuring during muscle development and cardiac disease (Bang et al., 2001). Overall, the biological function of obscurin–titin interactions has yet to be elucidated, although their association may provide a link between obscurin’s signaling domains and the physical localization of titin within the sarcomeric structure (Bang et al., 2001).

In *Drosophila* indirect flight muscles, super-resolution microscopy revealed that the insect titin ortholog Sls does not reach obscurin at the M-line and instead uses a titin-like molecule, projectin, to help bridge the gap in the A-band (Schueder et al., 2023). This organization is believed to stably anchor Sls to myosin filaments for effective force transduction without sarcomere rupture (Schueder et al., 2023). This hypothesized function is supported by the N-terminus of projectin, also containing homologous Ig domains typical of the I-band region of titin (Schueder et al., 2023). Given its inhomogeneous distribution across the A-band and M-line, it remains likely that other proteins stabilize this sarcomeric organization. Moreover, leg, jump, and larval muscles express longer variants of Sls that contain large PEVK domains within long I-bands (Schueder et al., 2023; Burkart et al., 2007), which may play an essential role in allowing force transduction across a broader range of movements. Though the role of obscurin during flight remains to be further studied, *Drosophila* obscurin appears to function mainly in myofibril assembly and is not directly connected to the titin-like molecules Sls and projectin.

### Obscurin/Obsl1 and myomesin interactions

Fukuzawa et al. (2008) discovered that obscurin forms a ternary complex with titin and myomesin, which anchors myosin filaments to the M-line. The N-terminal Ig1 domain of obscurin was found to directly bind the most C-terminal domain of titin, M10, which extends into the M-line, while the Ig3 domain of obscurin was simultaneously identified to bind the N-terminal linker region between the My4 and My5 domains of myomesin (Fukuzawa et al., 2008). To test whether obscurin’s Ig domains function independently in binding titin and myomesin, Fukuzawa et al. (2008) demonstrated that an obscurin fragment spanning Ig1–Ig3 was sufficient to target myomesin to titin and act as a crosslinker between them in cardiac muscle. Furthermore, transfection of exogenous fragments containing the relevant binding sites, such as obscurin Ig1 and Ig3, the myomesin My4–5 linker region, and titin M10 into neonatal rat cardiomyocytes, showed significant displacement of endogenous obscurin from the M-line and subsequent diffusion into the cytoplasm (Fukuzawa et al., 2008). The most pronounced effect was observed with transfection of Ig3 or My4–5, while the effects were less marked with Ig1 or M10 (Fukuzawa et al., 2008). This suggests that the interaction between obscurin and myomesin plays a more significant role in obscurin localization at the M-line than its interaction with titin. However, the incorporation of obscurin into the M-line depends critically on its direct binding with both titin and myomesin.

The Ig1 and Ig3 domains of Obsl1 showed functional homology in binding myomesin and titin (Fukuzawa et al., 2008). The largest Obsl1 isoform, Obsl1a, is predicted to contain 20 Ig and 1 FnIII domain identically organized as the N-terminal portion of obscurin (Benian and Mayans, 2015). Crystal fragments of obscurin and Obsl1 were mapped to interact with titin M10, forming chevron-shaped complexes similar to those of their Ig subunits, which associate through parallel packing of β-strands, yet with lower stability than other titin-containing complexes (Benian and Mayans, 2015). Benian and Mayans (2015) suggest that complexes with such low stability indicate the presence of additional binding partners that stabilize them. Nonetheless, similar obscurin/Obsl1 and titin M10 interactions remain mutually exclusive at different positions within the cell because Obsl1 lacks ankyrin-binding sites at its C-terminus for SR association (Pernigo et al., 2015). As a result, such obscurin interactions occur at the myofibril periphery, while Obsl1 interactions occur at the myofibrillar inner core (Pernigo et al., 2015). Pernigo et al. (2015) determined that M10–O1 interactions span 150 nm^2^.

Molecular dynamics simulations showed greater flexibility in M10:O1 than in the M10:OL1 complex due to its altered positioning of the βA′–βG hydrogen bond in the conventional intermediate set (I-set) subfamily of the Ig-fold, which is often found in muscle proteins (Pernigo et al., 2015; Otey et al., 2009). Analysis using the Pfam database identified this adjustment as an evolutionarily rare variant of the I-set, termed I*-set [69]. Unlike the conventional I-set where the βA′ strand hydrogen bonds with βG, the I*-set has βA′ interacting with βB (Pernigo et al., 2015). As multiple I*-set domains remain functionally uncharacterized, yet are situated in myomesin among other proteins, this variation likely allows intermolecular or intramolecular protein associations due to conformational fluctuations (Pernigo et al., 2015). This flexible interaction between obscurin and titin might serve as a regulatory checkpoint in M-line assembly, where fluctuations in its structure might cue sarcomeric restructuring versus turnover.

### Obscurin during titin assembly

Obscurin has also been found to affect titin assembly within skeletal myofibrils through its interactions with the scaffolding protein Ran binding protein-9 (RanBP9), which has been implicated in a host of signaling pathways and shown to interact with various proteins in different subcellular locations (Kontrogianni-Konstantopoulos et al., 2009). Bowman et al. (2008) found that adenovirus-mediated overexpression of obscurin’s RhoGEF domain significantly inhibited the ability of myotubes to integrate the N-terminal region of titin into Z-discs and subsequently disrupted the formation of both Z-discs and the A/I junction. They also found that overexpression of the RanBP9 region that binds obscurin’s RhoGEF domain resulted in similar disruptions to the integration of titin’s N-terminus into Z-discs (Bowman et al., 2008). Considering the direct interaction of obscurin’s RhoGEF domain with RanBP9 at a moderate binding affinity of 1.9 μM and the fact that both the RhoGEF domain and its binding region on RanBP9 are found to bind the Z-disc-adjacent region of titin independently, Bowman et al. (2008) suggested that obscurin and RanBP9 act together to integrate titin into the Z-disc. Remarkably, changes in the expression of obscurin’s RhoGEF domain or RanBP9’s obscurin-binding site did not affect the localization patterns of any other sarcomeric markers examined by this group, including that of titin epitopes concentrated at M- or I-bands (Bowman et al., 2008). This indicates that obscurin’s RhoGEF domain can cooperate with RanBP9 in a precise manner to incorporate the N-terminus of titin at Z-discs and that this process is independent of titin’s organization in other parts of the sarcomere (Bowman et al., 2008). Various regions of obscurin have demonstrated functions at disparate locations within the sarcomere, acting in conjunction with different binding partners, such as titin, myomesin, and RanBP9, to organize the overall structure of striated muscle tissue.

## RhoGEF domain of obscurin and small GTPase activation

The RhoGEF domain of obscurin has also been linked to a more traditional role in activating small GTPases, specifically RhoA and TC10 (also known as RhoQ) (Ford-Speelman et al., 2009; Coisy-Quivy et al., 2009). The first interactions between obscurin and a GTPase were identified in *C. elegans*, where the RhoGEF domain of UNC-89 was found to directly bind and activate Rho1, the RhoA equivalent in *C. elegans* (Qadota et al., 2008a). This study showed that the RhoGEF region of UNC-89 exhibits specific GDP exchange activity for Rho1 but not for other small GTPases, such as the *C. elegans* orthologs of Rac, RhoG, or Cdc42 (Qadota et al., 2008a). Additionally, partial RNAi-mediated knockdown of Rho1 displayed similar defects in the organization of myosin thick filaments as a loss-of-function mutant of UNC-89 lacking the RhoGEF domain, suggesting that this domain helps organize thick filaments by activating Rho1 GTPase (Qadota et al., 2008a). A later study by Ford-Speelman et al. (2009) discovered that the RhoGEF domain of obscurin directly binds and activates RhoA in mammalian skeletal muscle. As with UNC-89, other small GTPases, such as Rac1 and Cdc42, were not shown to interact with obscurin (Ford-Speelman et al., 2009). Using immunofluorescence, Ford-Speelman et al. (2009) found that obscurin’s RhoGEF domain co-localized with RhoA at M-lines in developing and mature skeletal muscle cells. However, overexpression of the RhoGEF domain prompted RhoA’s activation and redistribution from the M-line to the myoplasm and other sarcomeric structures, as well as changing the expression and activity of its downstream effector kinases (Ford-Speelman et al., 2009).

A similar change in the localization profile and activity of RhoA is observed in response to muscle injury caused by large-strain lengthening contractions, thus suggesting that obscurin-mediated activation of RhoA might be involved in muscle repair or hypertrophy (Ford-Speelman et al., 2009). This has interesting implications as obscurin is well-positioned to detect changes in the physical dimensions or integrity of sarcomeres and potentially activate intracellular and extracellular response pathways through its signaling domains. Meanwhile, an investigation by Coisy-Quivy et al. (2009) in human skeletal myocytes showed that obscurin’s RhoGEF domain directly binds and activates the small GTPase TC10. Experiments involving inhibition of TC10 demonstrated that this GTPase is crucial for the completion of myofibrillogenesis (Coisy-Quivy et al., 2009), thereby indicating that obscurin’s RhoGEF domain is likely also involved in sarcomere formation.

## Obscurin in non-muscle tissues

### Obscurin tissue expression and function

Since its discovery as a binding partner of titin, obscurin has been mainly studied within muscle, yet obscurin isoforms are also widely expressed outside this tissue (Ackermann et al., 2014). Using antibody staining in mice and rats, Ackermann et al. (2014) conducted a comprehensive investigation into the expression and subcellular localization of obscurin isoforms, ranging from 50 to 970 kDa, in non-muscle tissues, including the skin, brain, kidney, liver, spleen, and lung. In the skin, obscurin isoforms predominantly concentrate in epithelial cells, while in the kidney, they are present in various specialized endothelial and epithelial cells (Ackermann et al., 2014). They are also abundantly expressed in the liver, mainly at its external surface and within the hepatocytes’ cytoplasm (Ackermann et al., 2014). Obscurins are also widely expressed in the murine spleen, including in the B-cell follicle of the white pulp, suggesting a potential role in B-cell-related mechanisms such as antibody production (Ackermann et al., 2014). Obscurin isoforms are also located within the pleura of the lungs and the fibrous components of lung connective tissue (Ackermann et al., 2014). Across these tissue types, some obscurin isoforms show ubiquitous expression, while others are more cell-type-specific, often localizing to particular subcellular regions such as the nucleus, cytosol, and membrane (Ackermann et al., 2014). This indicates that different obscurin isoforms have both common and distinct functions in regulating cellular processes in various non-muscle tissues throughout the body.

A specific investigation into the non-muscle function of obscurin in zebrafish found obscurin isoform expression in the brain and retina, and experiments using morpholino-mediated silencing of the RhoGEF domain of obscurin showed that small GTPase activation by obscurin A is required for proper development of retinal neuroblasts through retinal lamination (Raeker et al., 2010). This proves that obscurin’s RhoGEF domain is involved in cellular differentiation in vertebrate neural tissue.

### Obscurin in cancer

A study examining the role of *OBSCN* mutations in several cancers found that obscurin is virtually absent in breast cancer cells but widely expressed in normal breast tissue (Perry et al., 2012). This phenomenon might stem from the loss of the obscurin RhoGEF domain and a subsequent decrease in RhoA signaling, leading to greater mobility of breast epithelial cancer cells through increased formation of tubulin-based projections, termed microtentacles (Perry et al., 2014). Another study found that depletion of giant obscurins in breast epithelial tissue leads to increased tumorigenicity and cancer metastasis by disrupting cell–cell adhesion and promoting mesenchymal behavior (Shriver et al., 2015). The disruption of cell–cell interactions was marked by changes in the expression of proteins associated with adherens junctions, such as N-cadherin and β-catenin, suggesting that giant obscurins stabilize proteins at cell–cell junctions in epithelial tissue (Shriver et al., 2015).

As *OBSCN* mutations remain elevated in tumorigenesis, it is plausible to consider the gene’s involvement as a tumor suppressor (Guardia et al., 2021). In breast cancer cells, epigenetic analysis of TCGA breast cancer datasets using the Wanderer tool identified substantial hypermethylation of the obscurin gene, leading to a significant decrease in transcription (Rajendran and Deng, 2017).

Recently, two variants of long non-coding RNAs were identified on the minus strand of *OBSCN*, termed *OBSCN-Antisense RNA 1/2* (*OBSCN-AS1/2*), which may have the potential to modify obscurin expression in breast cancer cells, although mutations have not yet been analyzed (Guardia et al., 2021). Analysis using the cBioPortal cancer genomics platform showed a positive correlation between *OBSCN-AS1* and *OBSCN* alterations in breast invasive carcinoma, suggesting that the lncRNA might influence obscurin expression (Guardia et al., 2021). It is possible that *OBSCN-AS1/2* lncRNAs function as epigenetic switches during cancer progression, but further research is needed to determine their exact function.

In summary, obscurin is an evolutionarily ancient, multimodular protein with a broad array of functions. A conserved role of invertebrate obscurin and its vertebrate homologs is their association with myofibrils at the M-line, likely through myosin, which helps ensure proper sarcomeric organization. Other conserved aspects of obscurin function are that obscurin connects myofibrils to the SR and mitochondria through cytoskeletal scaffolding and that its RhoGEF domain plays a role in signaling for muscle repair and maintenance. Many of these functions remain not fully understood, especially the roles of different isoforms and their differential localization during myofibril assembly, but new techniques such as super-resolution microscopy and better analysis of obscurin in model organisms promise further insights into these difficult problems.
